# Supplementary material for: Comparative analysis of shotgun metagenomics and 16S rDNA sequencing of gut microbiota in migratory seagulls
Source: PeerJ. 2023 Nov 3;11:e16394. doi: 10.7717/peerj.16394 (PMC10629391; doi:10.7717/peerj.16394)
Supplement: Supplemental Information 3 [file peerj-11-16394-s003.pdf]

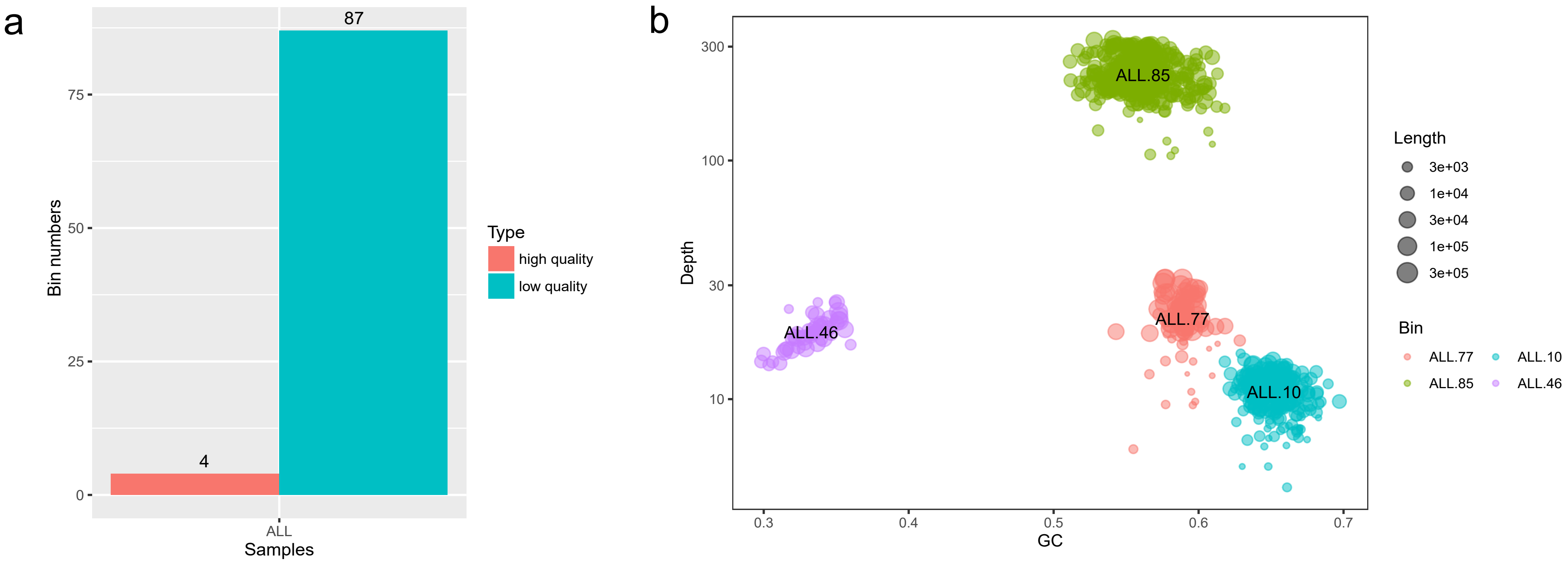

c

Bin	Contig num	Bin size(Mb)	Depth	Completeness	Contamination	Taxonmy	Tax_abundance
ALL.77	81	5.68	21.72	98.57	2.46	Bacteria; Proteobacteria; Gammaproteobacteria; Pseudomonadales; Pseudomonadaceae; Pseudomonas	0.99
ALL.85	372	3.68	227.84	92.32	1.36	Bacteria; Proteobacteria; Gammaproteobacteria; Enterobacterales; Erwiniaceae; Pantoea	0.98
ALL.10	253	2.54	10.71	85.8	1.75	Bacteria; Actinobacteria; Actinobacteria; Micrococcales; Micrococcaceae; Kocuria	0.93
ALL.46	48	0.92	19.1	81.68	1.31	Bacteria; Firmicutes; Bacilli; Lactobacillales; Lactobacillaceae; Lactobacillus	0.9
